# Adverse events in total knee arthroplasty: Results of a physician independent survey in 260 patients

**DOI:** 10.1186/1754-9493-4-12

**Published:** 2010-08-11

**Authors:** Stephan Kirschner, Jörg Lützner, Klaus-Peter Günther, Maria Eberlein-Gonska, Frank Krummenauer

**Affiliations:** 1 Department of Orthopedic Surgery University Hospital Carl Gustav Carus Medical Faculty of the Technical University of Dresden/Germany; 2 Quality Management University Hospital Carl Gustav Carus Medical Faculty of the Technical University of Dresden/Germany; 3 Department for Medical Biometry and Epidemiology Medical Faculty of Private University of Witten/Herdecke/Germany

## Abstract

**Purpose:**

Identification of all common and potentially avoidable adverse events is crucial to further improve the quality of medical care. The intention of the current study was to evaluate a standardized physician independent survey format on adverse events in total knee arthroplasty. The protocol for reporting adverse drug events following the International Conference of Harmonisation of technical requirements for registration of pharmaceuticals for human use (ICH) was adopted for adverse events occurring during surgical interventions.

**Material and methods:**

Data of a prospective sequential cohort trial introducing a clinical pathway for total knee arthroplasty was analysed. Reporting of adverse events was done by a physician independent study nurse using the modified ICH-Good Clinical Practice (GCP) format (Structure and Content of Clinical study reports - E3) in 260 patients. The adverse events were graded to their severity and their potential relation to surgical treatment.

**Results:**

A total of 55 patients (21%) suffered from an adverse event and 16 (6%) from a serious adverse event. In 38 patients' one adverse event occurred, 12 patients showed 2 adverse events and 5 patients suffered from a combination of an adverse and a serious adverse event. A serious adverse event alone occurred in 11 patients. The incidence of adverse events (Fisher p = 0.448) and serious adverse (p = 0.126) events showed no significant difference between the two cohorts. The most common adverse events were deep vein thrombosis (8% and 5%) followed by wound healing problems (1% and 0%) and haematoma (1% and 3%). A wide range of non surgical adverse events were recorded with low incidence levels.

**Conclusion:**

The use of the modified ICH-GCP format supports standardization of adverse event reporting. Routine assessment of adverse events by a study nurse revealed higher incidence rates of adverse events in total knee arthroplasty. We recommend the implementation of trained paramedical staff for the documentation of adverse events in routine clinical care.

## Introduction

Total knee replacement provides safe and effective treatment for patients suffering from end stage osteoarthritis[1]. Improvement in medical care offers total knee replacement to an increasing number of older patients even with severe comorbidities[2]. However, for the treatment of older and sicker patients an increased rate of medical complications has to be assumed,[3] but nevertheless the reported rate of adverse events is low, however, especially for the data reported to the national public external quality assuring system in Germany[4]. The reporting of adverse events in former papers on total joint replacement mainly focuses on major surgical adverse events like wound infection, fracture or loosening of the implant[5-10]. Therefore medical adverse events are likely to be underreported in these investigations [11]. In an evidence report on total knee arthroplasty [12] a more complete list of adverse medical as well as surgical events is published. Alfonso et al. [13] reviewed the non surgical complications after total hip and total knee arthroplasty and also used a more detailed listing of adverse events.

Beside this tendency in the orthopaedic literature there is an ongoing debate about the reliability of measurement and monitoring of adverse events[14]. Identification of all common and potentially avoidable adverse events is crucial to further improve the quality of medical care. Routine reporting by physicians identified fewer events than medical record review[15]. Therefore a different workflow to monitor adverse events in surgical patients may be advisable. A possible entry into an increased accuracy in monitoring may be the introduction of a trained study nurse or medical documentation assistant into the clinical routine surveillance system to thoroughly monitor adverse events in surgical patients undergoing total knee replacement; the well-established report instruction (Structure and Content of clinical study reports - E3) of the Good Clinical Practice (GCP) Guideline from the International Conference on Harmonisation of technical requirements for registration of pharmaceuticals for human use (ICH GCP[16] and ICH E3) may provide an efficient and standardized rationale for this additional stage in quality assessment.

## Patients and Methods

The intention of the current study was to propose and illustrate a standardized documentation and reporting assessment tool for (serious) adverse event patterns in Orthopaedic Surgery; furthermore the putative effect of the implementation of a physician independent survey on adverse events in total knee arthroplasty had to be evaluated. For that purpose data of a prospective sequential cohort trial introducing a clinical pathway for total knee arthroplasty was re-analysed. Two sequential cohorts were recruited with the aim of simultaneously estimating the process costs from the health care providers' perspective and the patient-related benefit of TKA. A total of 260 patients with the clinical indication for TKA were enrolled consecutively. Data on the present state was collected with a process documentation of the clinical course within a first cohort of 132 patients. The recorded data was used to derive and then implement an interdisciplinary clinical pathway on TKA; after a three months period of confirming this pathway proposal within clinical routine environments, a second independent cohort of 128 patients undergoing TKA was recruited and documented at the same hospital. Further sociodemographic details of both study cohorts are given in table 1. Furthermore all study patients were invited for a three months recall. The underlying prospective cohort design was ratified by the local Independent Ethics Committee by June 25^th ^2005. All patients gave informed consent to take part in this trial.

**Table 1:** Distribution characteristics for sociodemographic cofactors assessed in 132 patients, who underwent total knee arthroplasty (TKA) before implementation of a critical pathway on TKA, versus 128 patients, who underwent TKA after path implementation

	Before pathway implementation (n = 132)	After pathway implementation (n = 128)
***Age: median and range [years]***	68 (43 - 88) years	70 (46 - 85) years

***Females***	64%	56%

***living alone***	32%	28%

***under employment***	8%	9%

***graduate***	20%	17%

### Surgical procedure

The study population consisted of patients with primary or secondary knee osteoarthritis grade 3 and 4 according to Kellgren and Lawrence[17]. Surgery was performed in nearly all patients under regional anaesthesia and a tourniquet was applied after admission of preoperative antibiotic prophylaxis (third generation cephalosporine). Through a straight skin incision and a medial parapatellar approach a cemented Natural Knee II (Fa. Zimmer, Germany) total knee arthroplasty without resurfacing of the patella was implanted. All patients undergoing total knee replacement were graded to be at high risk for deep venous thrombosis. All patients received Arixtra 1 × 1 s. c. for 6 weeks. Postoperatively all patients were allowed for immediate full weight bearing. After discharge from the hospital the patients were transferred to an inpatient rehabilitation unit for additional three weeks.

### Monitoring of adverse events and data collection

All data collection preoperatively and at follow-up was done by a trained medical documentation assistant. Functional ability and health-related quality of life were assessed by means of appropriate instruments (WOMAC[18], EQ-5D[19] and Knee Society Knee Score)[20]. For monitoring of adverse and serious adverse events the definitions of the ICH GCP were adapted for surgical interventions and implemented as described below. The original wording is given in square brackets followed by the new text in italic letters.

*Definition of adverse events (AE): *any unexpected medical occurrence in a patient or clinical investigation subject [administered a pharmaceutical product] *undergoing surgical treatment*, which does not necessarily have a causal relationship with this treatment. An adverse event (AE) can therefore be any unfavourable and unintended sign (including an abnormal laboratory finding), symptom, or disease temporally associated with [the use of a medicinal (investigational) product] *surgical treatment*, whether or not related to [the medicinal (investigational) product] *surgical treatment*.

*definition of serious adverse events (SAE): *any unexpected medical occurrence [at any dose] *in the operative period*, which resulted in death, went life-threatening, required inpatient hospitalization or prolongation of existing hospitalization, or resulted in persistent or significant disability/incapacity.

Averse events and serious adverse events were recorded at time of clinical appearance or at time of first available information to the Orthopaedic surgery department. In any case of a suspected deep vein thrombosis the patients were admitted to the university vascular centre and further sonographic diagnostics were carried out. There is no general screening for DVT in patients undergoing TKA. A medical documentation assistant interviewed all patients at follow up and actively screened any adverse event possible after knee arthroplasty. In order to grade the adverse events according to their severity and their potential relation to surgical treatment all (S)AE reports by the medical documentation assistant were reviewed by the coordinating clinical investigator. Almost all patients suffering from an adverse event were consulted by a specialist. The (S)AE events were graded "surely, possibly, probably, non related". Only in the case of a undoubtedly pre-existing disease like a Hyperhomocystinemia the (S)AE was graded "non related". In all other cases a possible relation to surgical treatment was considered. At the time of the three months follow-up investigation the impact of the adverse event on mid-term outcome after total knee replacement was estimated. No structured interviews were done afterwards.

### Statistical analysis

The descriptive analysis of this embedded quality investigation was based on two-sided Fisher tests at a local 5% significance level to compare the cumulative incidences of adverse events and serious adverse events between the two sequential cohorts. The observed (serious) adverse events were listed according to the ICH report form.

To further investigate possible factors contributing to the observed (serious) adverse event patterns a severity score on the individual adverse event patterns was constituted: The appearance of two adverse events or a combination of an adverse event together with a serious adverse event was hypothesized to be a more severe impairment on individual health than a single adverse events or no adverse event at all. The severity score counted one point for every adverse event and two points for serious adverse events. Description of this score was based on relative frequencies. Furthermore a multiple logistic regression model was fitted for this score by means of a forward selection model and Likelihood Ratio tests at a local 5% significance level: A total score of 2 points or more was considered "severe" and applied as a surrogate endpoint of the overall (S)AE patterns; age at time of surgery, the patients' individual comorbidity and complexity level (pccl, derived form the DRG calculation), gender, educational level, WOMAC index before surgery and cut suture time were introduced into the model as possible explaining factors, the patients' respective cohort - before versus after pathway implementation - was furthermore included.

All numerical evaluations were based on the software SPSS^® ^(release 17.0 for Windows^®^).

## Results

A total of 55 patients (21%) suffered from an adverse event and 16 (6%) from a serious adverse event. The improvement in terms of patient related outcome measures for WOMAC, EQ-5 D and Knee Society Knee and Function Score is given in table 2. In 38 patients' one adverse event occurred, 12 patients showed 2 adverse events and 5 patients suffered from a combination of an adverse and a serious adverse event. A serious adverse event alone occurred in 11 patients. In the entire cohort at total of 68 adverse events and 16 serious adverse events were recorded, 30 + 5 of which reported form the first cohort (23% + 4%) and 37+11 from the second cohort (29% + 11%) after clinical pathway implementation. The incidence of adverse events (Fisher p = 0.448) and serious adverse (p = 0.126) events showed no significant difference between the two cohorts. The most common adverse events were deep vein thrombosis with an occurrence rate of 8% and 5% for the lower leg in the respective cohorts. Further surgical adverse events like wound healing problems (1% and 0%) and haematoma (1% and 3%) were observed less frequently. Falls (2%) were only reported for the second cohort. Further adverse events were reported at a lower level of incidence (Table 3).

**Table 2:** Medians and quartiles for the total WOMAC osteoarthritis index, EQ-5 D [%, 100% = optimum rating] and Knee Society Knee and Function Score before and three months after total knee arthroplasty (TKA)

	Before pathway implementation (n = 132)	After pathway implementation (n = 128)
***EQ-5D***		

*preoperative [%]*	**45% **(35 - 50%)	**50% **(31 - 50%)

*postoperative [%]*	**75% **(60 - 85%)	**75% **(60 - 80%)

***WOMAC***		

*preoperative [%]*	**41% **(32 - 48%)	**44% **(36 - 52%)

*postoperative [%]*	**83% **(68 - 91%)	**82% **(70 - 91%)

***Knee Society***		

***Knee Score***		

*preoperative [pt]*	**43 **(29-54)	**46 **(37-56)

*postoperative [pt]*	**87 **(69-94)	**85 **(65-92)

***Knee Society Function Score***		

*preoperative [pt]*	**50 **(40-60)	**55 **(45-60)

*postoperative [pt]*	**60 **(50-75)	**65 **(55-76)

**Table 3:** Adverse event and serious adverse event counts, percentages given in brackets, for 132 patients, who underwent total knee arthroplasty (TKA) before implementation of a critical pathway on TKA, versus 128 patients, who underwent TKA after path implementation

	Before pathway implementation	After pathway implementation	Total
adverse event	n = 132	n = 128	n = 260
Generalised seizure due to local anaesthetic intoxication	0 (0)	1 (1)	1 (0)

DVT (lower leg)	10 (8)	6 (5)	16 (6)

DVT (knee)	4 (3)	2 (2)	6 (2)

DVT (thigh)	0 (0)	1 (1)	1 (0)

Haematoma	1 (1)	3 (2)	4 (2)

Wound healing problems	1 (1)	0 (0)	1 (0)

Stiff knee	0 (0)	1 (1)	1 (0)

Gastrointestinal bleeding	0 (0)	3 (2)	3 (1)

Dekubital ulcer	1 (1)	2 (2)	3 (1)

Postoperative cognitive disorder	1 (1)	0 (0)	1 (0)

Speech disturbance and Hyperkinesia in Parkinson disease	2 (2)	1 (1)	3 (1)

Postoperative hypertonia	1 (1)	3 (2)	4 (2)

Cardiac blocks	2 (2)	2 (2)	4 (2)

Diarrhoea	1 (1)	1 (1)	2 (1)

Obstipation	0 (0)	1 (1)	1 (0)

Erysipel	1 (1)	0 (0)	1 (0)

Exanthema	1 (1)	3 (2)	4 (2)

Otolaryngology consultation*	2 (2)	0 (0)	2 (1)

Dentist consultation*	1 (1)	0 (0)	1 (0)

Urinary tract infection	0 (0)	2 (2)	2 (1)

Urologic consultation*	1 (1)	2 (2)	3 (1)

Fall	0 (0)	2 (2)	2 (1)

Posterior collum lesion (myelon)*	0 (0)	1 (1)	1 (0)

Hyperhomocystinemia*	0 (0)	1 (1)	1 (0)

Total AE	30 (23)	38 (29)	68 (26)

**serious adverse event**			

Death during study period, unrelated	1 (1)	1 (1)	2 (1)

Resuscitation (cardiological cause)	1 (1)	0 (0)	1 (0)

Pulmonary embolism	1 (1)	0 (0)	1 (0)

Revision for infection	1 (1)	3 (2)	4 (2)

Revision for haematoma	1 (1)	0 (0)	1 (0)

Closed mobilisation	0 (0)	3 (2)	3 (1)

Opticusneuropathia	0 (0)	1 (1)	1 (0)

Myocardial infarction	0 (0)	1 (1)	1 (0)

Central vein thrombosis eye	0 (0)	1 (1)	1 (0)

Subcutaneous infection belly (related to DVT prophylaxis)	0 (0)	1 (1)	1 (0)

Total SAE	5 (4)	11 (9)	16 (6)

* Adverse events unrelated to surgical treatment

In cohort 1 (before pathway implementation), a total of 10 patients (8%) showed a severity adverse event score of 2 or more, in cohort 2 (after pathway implementation) a total of 17 patients (13%). The logistic regression model could only identify the individual pccl (LR p < 0.001) and the a patient's cohort designation (LR p = 0.036) as significantly associated with this endpoint; neither age at time of surgery (LR p = 0.062) nor gender (LR p = 0.106) nor educational level (LR p = 0.096) nor cut/suture time (LR p = 0.366) nor WOMAC (LR p = 0.344) before surgery were found significantly associated with the occurrence of a severity score of 2 points and more.

## Discussion

Improvement in medical care extends the life expectancy of our patients and secondary increases the need for total joint replacement[21]. For surgical procedures with a high volume the introduction of clinical pathways was promoted[22]. The quality of medical care can be improved through the use of clinical pathways embedded within a quality management system by continuously measuring and evaluating process and outcome indicators[23]. Especially for elder patients this systematic and structured improvement process could be beneficial and illustrates again the relevance of the PDCA-cycle of Walter Deming. Therefore a vital part of continuous quality assurance is the monitoring of adverse events and to evaluate whether these were avoidable[24]. University hospitals are more likely to treat patients at higher age and with more complex comorbidities. Therefore a higher rate for adverse events has to be assumed in these institutions.

The rates for adverse events (21%) and serious adverse events (6%) in our patients appear relatively high when compared to the official data of the national external quality assurance system in Germany[4]. In this nation wide database for example the average incidence of general medical complications was reported to be 2% with a reference limit of 5%. Data published by Parvizi et al. [3] with 21% minor complications and 5% major complication, however, match better to our observations. Similar results were found by Frosch et al.[25]. Alfonso [13] provided an extensive review of the non orthopaedic literature and confirmed a broad range of non surgical adverse events in patients undergoing total joint replacement. The importance to use accepted definitions was highlighted by Bruce et al.[14]. Especially in the context of suspected wound infection he found only little evidence for systemic measurement and monitoring of surgical wound. The strict definition for persisting wound drainage by Parvizi [3] as any secretion from the wound 48 hours post surgery is easy to use. In case of postoperative haematoma or suspected wound infection active treatment with revision of the wound is recommended. Timing of the intervention is important for further prognosis[26]. If in doubt a revision should be undertaken. The clear and comprehensible definition makes it easier for the patient to understand the situation and to agree to further surgery. We carried out 4 revisions for suspected early infection and one for haematoma.

Falls of patients occurring after surgical procedures performed under regional anaesthesia are reported in the literature[27]. We observed these only in the second cohort of our patients. During pathway implementation no changes concerning the use of regional anaesthesia were made. In contrast the patients of the second cohort were offered to attend further information about the disease, the operation and the pain management with regional anaesthesia thereafter. The increased rate of documented falls may be attributable to closer monitoring of our patients. Higher age as well as multiple comorbidities are a documented risk factors for the occurrence of adverse events[28,29]. We were able to confirm the impact of comorbidities expressed in elevated pccl level in our patients.

Treatment of patients suffering from severe Parkinson disease with brain stimulators is a focus of the neurological and neurosurgical department at our university[30]. In the postoperative period these patients are closely monitored by these specialists. The reported adverse events in these patients may be an effect of this intensive care. None of these patients showed worsened mental state at the time of discharge.

Among the reported serious adverse results the opticusneuropathia as well as the thrombosis of the central eye vein is unrelated to treatment to our understanding. Major surgical adverse events like revisions for haematoma or suspected infection are similar in both cohorts. Treatment for decreased motion after total knee arthroplasty was more aggressive in the second cohort as part of the clinical pathway[31]. In consequence we observed three closed manipulations only in the second cohort. There was an increase in serious adverse events for cohort II, but the difference in incidence rates were not found significant at all.

There are some limitations of the current study. We reported the incidence of adverse events for a clinical trial by means of a rather small series of 260 patients only. Larger numbers of patients could probably identify more influencing factors like age for the occurrence of adverse events and identify additional (S)AEs with smaller incidences than being observable by means of the recent sample size. As the investigation was performed at a tertiary referral centre our cases might represent a selected cohort with a higher percentage of elderly, more extended use of anticoagulative drugs and higher prevalence of comorbidities. Generalization of the above quantitative results is therefore limited; however, bearing the primary intention of proposing and illustrating a standardized reporting system, the pilot investigation ended with a positive conclusion in confirming the feasibility of the proposal demonstrated here.

In summary, further improvement in the quality of medical care depends on full recognition of adverse events. Routine assessment of adverse events by a trained medical documentation assistant or study nurse could lead to a more complete documentation and therefore to a higher rate of reported adverse and serious adverse events. The independency of study nurse assessment from physicians and the nursing team seem to be useful to achieve a more constructive discussion culture as highlighted by de Vries[32]. We therefore recommend the implementation of trained paramedical staff for the documentation of adverse events in routine clinical care.

## Competing interests

The authors have no commercial or political interests in the methodological or medical details presented in this paper. The investigation was granted by Zimmer^® ^GmbH Deutschland: the study coordinator position of Ms Claudia Wolf was sponsored by a company grant.

## Authors' contributions

SK Study design, Obtaining of funding, statistical analysis and interpretation of the data, drafting and revision of the article.

JL Contribution to the study design, discussion of the results, technical and logistic support.

KPG administrative, technical, and logistic support.

MEG Support in study design with special reference to quality management.

FK Study design, Obtaining of funding; statistical expertise; analysis and interpretation of the data; administrative, technical and logistic support.

All authors read and approved the final manuscript.

## References

[B1] NIH Consensus Statement on total knee replacement December 8-10, 2003 J Bone Joint Surg Am 2004 86-A 1328 1335 15173310 10.2106/00004623-200406000-00031

[B2] Raja SN Haythornthwaite JA Anesthetic management of the elderly: measuring function beyond the immediate perioperative horizon Anesthesiology 1999 91 909 911 10.1097/00000542-199910000-00006 10519489

[B3] Parvizi J Mui A Purtill JJ Sharkey PF Hozack WJ Rothman RH Total Joint Arthroplasty: When Do Fatal or Near-Fatal Complications Occur? J Bone Joint Surg Am 2007 89 27 32 10.2106/JBJS.E.01443 17200306

[B4] Ungeplante Folgeoperation wegen Komplikation - BQS Report http://www.bqs-online.com/public/bqsfp/qifp/2006/knie_schlitten_erstimp/10 accessed on 25.3.2010

[B5] Mont MA Mears SC Jones LC Rajadhyaksha AD Krackow AM Bawa M Hungerford DS Is coding of diagnoses, comorbidities, and complications in total knee arthroplasty accurate? J Arthroplasty 2002 17 767 772 10.1054/arth.2002.33549 12216032

[B6] Schai PA Thornhill TS Scott RD Total knee arthroplasty with the PFC system. Results at a minimum of ten years and survivorship analysis J Bone Joint Surg Br 1998 80 850 858 10.1302/0301-620X.80B5.8368 9768897

[B7] Ranawat AS Ranawat CS Elkus M Rasquinha VJ Rossi R Babhulkar S Total knee arthroplasty for severe valgus deformity J Bone Joint Surg Am 2005 87 Suppl 1 271 284 10.2106/JBJS.E.00308 16140800

[B8] Ranawat CS Flynn WF Jr Deshmukh RG Impact of modern technique on long-term results of total condylar knee arthroplasty Clin Orthop Relat Res 1994 131 135 7994951

[B9] Hofmann AA Heithoff SM Camargo M Cementless total knee arthroplasty in patients 50 years or younger Clin Orthop Relat Res 2002 102 107 10.1097/00003086-200211000-00018 12439246

[B10] Wright RJ Sledge CB Poss R Ewald FC Walsh ME Lingard EA Patient-reported outcome and survivorship after Kinemax total knee arthroplasty J Bone Joint Surg Am 2004 86-A 2464 2470 15523019 10.2106/00004623-200411000-00016

[B11] Goldhahn S Sawaguchi T Audige L Mundi R Hanson B Bhandari M Goldhahn J Complication reporting in orthopaedic trials. A systematic review of randomized controlled trials J Bone Joint Surg Am 2009 91 1847 1853 10.2106/JBJS.H.01455 19651940

[B12] Kane RL Saleh KJ Wilt TJ Bershadsky B Cross WW III MacDonald RM Rutks I Total knee replacement Evid Rep Technol Assess (Summ) 2003 1 8 15160414 PMC4781011

[B13] Alfonso DT Toussaint RJ Alfonso BD Strauss EJ Steiger DT Di Cesare PE Nonsurgical complications after total hip and knee arthroplasty Am J Orthop 2006 35 503 510 17152971

[B14] Bruce J Russell EM Mollison J Krukowski ZH The measurement and monitoring of surgical adverse events Health Technol Assess 2001 5 1 194 11532239 10.3310/hta5220

[B15] Marang-van de Mheen PJ van Hanegem N Kievit J Effectiveness of routine reporting to identify minor and serious adverse outcomes in surgical patients Qual Saf Health Care 2005 14 378 382 10.1136/qshc.2004.013250 16195574 PMC1744069

[B16] ICH E3 Structure and Content of Clinical Study Reports http://www.ema.europa.eu/docs/en_GB/document_library/Scientific_guideline/2009/09/WC500002832.pdf

[B17] Kellgren JH Lawrence JS Atlas of standard radiographs of arthritis 1963 Oxford: Blackwell Scientific Publications

[B18] Bellamy N Buchanan WW Goldsmith CH Campbell J Stitt LW Validation study of WOMAC: a health status instrument for measuring clinically important patient relevant outcomes to antirheumatic drug therapy in patients with osteoarthritis of the hip or knee J Rheumatol 1988 15 1833 1840 3068365

[B19] Brooks R EuroQol: the current state of play Health Policy 1996 37 53 72 10.1016/0168-8510(96)00822-6 10158943

[B20] Insall JN Dorr LD Scott RD Scott WN Rationale of the Knee Society clinical rating system Clin Orthop Relat Res 1989 13 14 2805470

[B21] Iorio R Robb WJ Healy WL Berry DJ Hozack WJ Kyle RF Lewallen DG Trousdale RT Jiranek WA Stamos VP Orthopaedic surgeon workforce and volume assessment for total hip and knee replacement in the United States: preparing for an epidemic J Bone Joint Surg Am 2008 90 1598 1605 10.2106/JBJS.H.00067 18594111

[B22] Coffey RJ Richards JS Remmert CS LeRoy SS Schoville RR Baldwin PJ An introduction to critical paths Qual Manag Health Care 2005 14 46 55 15739581 10.1097/00019514-200501000-00006

[B23] Barbieri A Vanhaecht K Van Herck P Sermeus W Faggiano F Marchisio S Panella M Effects of clinical pathways in the joint replacement: a meta-analysis BMC Med 2009 7 32 10.1186/1741-7015-7-32 19570193 PMC2715423

[B24] de Vries EN Ramrattan MA Smorenburg SM Gouma DJ Boermeester MA The incidence and nature of in-hospital adverse events: a systematic review Qual Saf Health Care 2008 17 216 223 10.1136/qshc.2007.023622 18519629 PMC2569153

[B25] Frosch P Decking J Theis C Drees P Schoellner C Eckardt A Complications after total knee arthroplasty: a comprehensive report Acta Orthop Belg 2004 70 565 569 15669457

[B26] Mont MA Waldman B Banerjee C Pacheco IH Hungerford DS Multiple irrigation, debridement, and retention of components in infected total knee arthroplasty J Arthroplasty 1997 12 426 433 10.1016/S0883-5403(97)90199-6 9195319

[B27] Feibel RJ Dervin GF Kim PR Beaule PE Major complications associated with femoral nerve catheters for knee arthroplasty: a word of caution J Arthroplasty 2009 24 132 137 10.1016/j.arth.2009.04.008 19553071

[B28] Kreder HJ Berry GK McMurtry IA Halman SI Arthroplasty in the octogenarian: quantifying the risks J Arthroplasty 2005 20 289 293 10.1016/j.arth.2004.09.024 15809944

[B29] SooHoo NF Lieberman JR Ko CY Zingmond DS Factors predicting complication rates following total knee replacement J Bone Joint Surg Am 2006 88 480 485 10.2106/JBJS.E.00629 16510811

[B30] Storch A Hofer A Kruger R Schulz JB Winkler J Gerlach M New developments in diagnosis and treatment of Parkinson's disease--from basic science to clinical applications J Neurol 2004 251 Suppl 6 VI/33 VI/38 10.1007/s00415-004-1608-4 15675723

[B31] Bhave A Mont M Tennis S Nickey M Starr R Etienne G Functional problems and treatment solutions after total hip and knee joint arthroplasty J Bone Joint Surg Am 2005 87 Suppl 2 9 21 10.2106/JBJS.E.00628 16326719

[B32] de Vries EN Ramrattan MA Smorenburg SM Gouma DJ Boermeester MA The incidence and nature of in-hospital adverse events: a systematic review Qual Saf Health Care 2008 17 216 223 10.1136/qshc.2007.023622 18519629 PMC2569153

